
JOURNAL INFORMATION
==============================
NLM Title Abbreviation: Eur Psychiatry
Journal ID: Eur Psychiatry
NLM Journal ID: 9111820
Journal ID: EPA

European Psychiatry

ISSN: 0924-9338
EISSN: 1778-3585
Publisher: Cambridge University Press

ARTICLE INFORMATION
==============================
PMCID: PMC9412720
DOI: 10.1192/j.eurpsy.2020.12
Article ID: S0924933820000127
Article version: 1
Subjects: Abstracts

Workshop

Publication date: 2020 Jul
Electronic publication date: 2020 Sep 4
Volume: 63
Issue: Suppl 1
Issue title: Abstracts of the 28th European Congress of Psychiatry
First page: S620
Last page: S628
Copyright: © The Author(s) 2020
Copyright year: 2020
Copyright holder: The Author(s)
License: This is an Open Access article, distributed under the terms of the Creative Commons Attribution licence (http://creativecommons.org/licenses/by/4.0/), which permits unrestricted re-use, distribution, and reproduction in any medium, provided the original work is properly cited.
License URL: https://creativecommons.org/licenses/by/4.0/

Page count: 9

==============================

### Acute short-lived psychotic disorders: clinical, nosological and socio-cultural aspects

### Abstracts

#### W004

#### Clinical, demographic characteristics and outcome of acute and transient psychotic disorder in Ibadan, Nigeria

O. Esan

**Background:** Although ICD-10 "acute and transient psychotic disorders" (ATPDs) are more common in developing countries than in developed ones, little is known about factors associated with the course and outcome of these conditions. This study examines the predictive strength of baseline clinical and demographic variables and the short-term outcome of ATPDs in a developmental country setting.
**Method:** Retrospective study of all patients with a first episode ATPD attending the University College Hospital, Ibadan, Nigeria between 2006 and 2011, and who had at least 1-year follow-up information available. Psychopathological symptoms were rated using the 5-factor model of the Positive and Negative Syndrome Scale (PANSS). Multivariate analyses were conducted to identify clinical and demographic outcome predictors.
**Results:** Of the 52 patients with ATPDs (59.6% women; mean age 27.5 years), 33 (63.5%) did not develop another diagnosis on average over 4.8 years. Patients featuring polymorphic psychotic symptoms were less likely to change diagnosis than those with the acute schizophrenia-like presentation, who instead tended to progress mainly to schizophrenia in subsequent episodes. No baseline variable was found to be significantly associated with temporal stability of ATPDs. Multivariate analyses revealed that excitement/hostility was the only PANSS factor predicting diagnostic transition.
**Conclusion:** These findings suggest that the diagnostic consistency of ATPDs is relatively high in Ibadan, and argue for prognostic differences among the ATPD subtypes.

### Early intervention/early detection in psychosis in real-life practice: different models across Europe

#### W009

#### Implementing early detection of psychosis in France: the potential of adolescents' houses

V. Laprevote[1]*, T. Mignot[2], F. Bernardin[1] and R. Schwan[3]

EUROPEAN PSYCHIATRIC ASSOCIATION

Despite the delay accumulated in their implementation, France actually develops a growing interest in early detection (ED) and early intervention (EI) services. The French network of Maisons des Adolescents (Adolescents' Houses) offer various advantages for ED/EI. Adolescent's houses are dedicated to prevention, health promotion and orientation to specific care of young people between 11 and 25. French government has developed a territorial coverage of these centres since 2005, and 111 Adolescents' Houses are actually implemented all over the country. The CLIP (Centre de Liaison et d'Intervention Précoce) has been developed in the Adolescents' House of Nancy (East of France). This centre offers evaluation and care for young people with an

At Risk Mental State for Psychosis or a non medicated First Episode of Psychosis. Such a partnership provides clear benefits for ED/EI approach, but has to be adapted to Adolescents' House constraints.

### W012

#### First episode psychosis integrative treatment: a 20-year experience in Tallinn, Estonia

K. Peebo

Existing guidelines for first episode psychosis recommend identification and timely treatment of first-episode psychosis patients through specific early intervention programmes. Most guidelines also highlight the importance of family intervention to prevent relapse. The first episode psychosis integrative treatment programme in Tallinn, Estonia was established in 1997. The programme stems from the need-adapted model developed by Yrjö O. Alanen in Finland. The integrative family-centered team approach can be described by different levels: integration of different treatment modalities, inpatient and outpatient treatment, different specialities in a multiprofessional team and integration of multilevel expertise involving families. The efficacy of the integrative treatment model was investigated in a two-year follow-up. The study showed that patients in the programme had improved quality of life and employment rates after two years. This model is being used today with the same core features and with some new approaches included over the years. The aim of this presentation is to provide an introduction to the integrative family-centered team model and the psychotherapeutic and psychosocial interventions used in The First Episode Psychosis Integrative Treatment Department in Tallinn Psychiatry Clinic.

## Assisted suicide in the elderly

### W023

#### Media and suicidal behaviour

U. Hegerl[1,2]

Three aspects will be discussed which are considered relevant for preventing suicidal behaviour: 1) Media coverage of suicides can induce not only acute increases in suicides via the well-known Werther-effect but also long-lasting changes in suicidal behaviour. Increasing cognitive availability for certain suicide methods is a possible explanation for this long-lasting effect (Hegerl et al 2013, J Affect Dis). 2) Rapid propagation of informations about certain suicide methods (e.g. carbomonoxide via charcoal burning) influences the choice of suicide methods and the lethality of suicidal acts with major impact on suicide rates. It will be discussed whether increases in suicide rates for younger people and females observed in some countries are explained by an increased lethality of suicidal intoxications due to social media effects. 3) Suicide preventive campaigns are likely to improve help seeking behaviour but also to lower threshhold by normalizing suicidal behaviour. The balance between these two effects is unclear and will be discussed.

## Psychotherapy training in Europe

### W024

#### Trainees are motivated but have difficulty to find resources

T. Gargot

Evidence for efficacy of psychotherapies growing and the inclusion of such therapies in international guidelines for treatment of psychiatric disorders is increasing as well. Here, we present a cross sectional survey conducted by the EFPT psychotherapy working group in 2013-2015 and published in European Psychiatry journal. 574 professionals from 22 European countries answered online. 92% of trainees considered psychotherapy as important for their professional identity. 90% would like to practice psychotherapy after their training. 96 % would train for free. 50 % intend to spend up to 5 % of their salary for psychotherapy training. Psychoanalysis, CBT and Systemic Therapy were the most popular schools of psychotherapy. For most trainees, only theoretical lectures are mandatory. Whereas training in psychotherapy is recommended by UEMS as a mandatory component of curriculum, most trainees don't receive any or need to pay for it. Recommendations of UEMS are reported to be implemented in 22% of respondents' country of origin. Less than 50 % of participants received a training in psychotherapy. 33 % undertook psychotherapy training on their own initiative. Whereas more than 1.7 hours of supervision per month are recommended by UEMS. 50 % don't get any hour during the month. Related survey are conducted at the moment by the World Psychiatrist Association and the EFPT Child and Adolescent working group to confirm and precise this data. From this assessment, EFPT and EPA proposed different initiatives to improve this training: online ressources e-training, MOOC, journal club, psychotherapy guidebook. http://efpt.eu/wp-content/uploads/2019/01/psychotherapy.pdf

### W026

#### EFPT psychotherapy guidebook: the story

T. Koutsomitros

There are so many kinds of psychotherapies out there and what better way to explain and discuss them in a peer-to-peer relationship than a trainee exchanging ideas about experiences in the particular therapy with a fellow trainee.
It all started in London in 2014, when some colleagues from our EFPT group started to discuss the idea of creating a free guidebook on Psychotherapy written by trainees for trainees. The idea travelled across Europe along with the members of our group. It expanded and evolved year after year, from forum to forum, when finally, the time had come to return to the UK, in Bristol this time, in 2018 with its first edition of our free EFPT psychotherapy guidebook. This year we also updated a mobile friendly version of it on a web authoring platform. The 1st edition contained 12 chapters. Our psychotherapy working group has expanded and together with the increase in the number of our members, we managed to double the number of chapters of our psychotherapy guidebok.
The goal is for each chapter to be written by trainees who have had experience or interest in that particular psychotherapy. There are many psychotherapies that we have not included yet in our guidebook that are looking for authors, contributors! Let's all put some effort to make our next versions of our Psychotherapy guidebook a valuable gift to our younger colleagues who are choosing their path and to all of us who never stop looking for new challenges and knowledge.

### W028

### EPA online course about motivational interviewing: present and future perspective

P. Browne

An EPA survey in 2018 asked members about desirable topics for future courses and identified Motivational Interviewing (MI) as the second most popular option, with 11% of 693 respondents choosing it. Consequently the EPA Committee on Education chose MI as the topic of their second online video course, following the successful 'Introduction to CBT' online course produced in 2018. The new course, titled 'Introduction to Motivational Interviewing', was made under the leadership of NHS consultant psychiatrist Dr Guy Undrill who is a member of the MI Network of Trainers. The videos also feature three of his current psychiatry trainees and an actor, which allowed the teaching style to reflect MI principles and made it possible to include roleplay scenarios that brought the topic to life. The course was filmed in January 2019 and the final version was released in June 2019 accompanied by a discussion forum in which the makers of the course answered questions about MI and how to use it in psychiatry. In this workshop we will explain what MI is, why it is suitable for an online course, and how the course was produced. We will also reflect on the initial feedback regarding the effectiveness of the course and think about the next steps for improving psychotherapy training for European psychiatrists.

## Antipsychotics and antidepressants dosing. Focus on elderly patients

### W029

### Recommendations for dosing antipsychotics in schizophrenia

I. Bitter

The significant majority of patients with schizophrenia benefit from long term (maintenance) antipsychotic treatment (1), however the doses used in the practice are often higher, than recommended based on PET (2) and clinical studies (3). The two major sources of overdosing antipsychotics in patients with schizophrenia have been: 1. Lack of high quality studies about dose-efficacy relationship for antipsychotics in schizophrenia until the 1990s or even until recently. Studies with improved design, including plasma level and SPECT/PET as well as meta-analytic studies helped in understanding the relationship between dose of antipsychotics and clinical effects. 2. The frequent use of antipsychotic polypharmacy in schizophrenia (4). Polypharmacy has been widely practiced, often leading to high cumulative doses of antipsychotics, however the rigid rejection of antipsychotic polypharmacy by guidelines prevented the support of appropriate research in this field. Based on current evidence the following recommendations can be proposed: 1. Better training in psychopharmacology, including dosing of antipsychotics. 2. Supporting adequate research in antipsychotic polypharmacy in schizophrenia. References 1. Herold, R., Szekeres, G., & Bitter, I. (2017). Continuous maintenance antipsychotic treatment in schizophrenia. Psychiatria Hungarica, 32(3), 296-306. 2. Tauscher, J., Kapur, S. (2001). Choosing the right dose of antipsychotics in schizophrenia. CNS Drugs, 15(9), 671-678. 3. Leucht, S., et al. Dose-Response Meta-Analysis of Antipsychotic Drugs for Acute Schizophrenia. American Journal of Psychiatry 2019 Dec 16: appiajp201919010034. doi: 10.1176/appi.ajp.2019.19010034. [Epub ahead of print] 4. Tiihonen, J. et al. (2019). Antipsychotic Polypharmacy vs Monotherapy With Psychiatric Rehospitalization Among Adults With Schizophrenia. JAMA Psychiatry, 76(5), 499-507.
**Disclosure:** Dr. Bitter reports personal fees from Angelini, Eli Lilly,Janssen/Janssen-Cilag and Gedeon Richter outside of the submitted work; grants from the European Commission and European Group into Research in Schizophrenia.

### W032

### The use of antidepressants in elderly patients with various psychiatric disorders

F. Bouckaert

Depression is one of the most common mental health problems in the elderly and is associated with a significant burden of illness affecting patients, their families and communities. Selecting, adjusting, and changing antidepressants in the elderly in a sequential way has shown response and remission rates similar to those of younger adults. However, antidepressants may be less effective in older patients because they have a greater burden of somatic disorders (including white matter lesions) and because their physicians have a tendency to prescribe suboptimal doses. Physicians are also increasingly prescribing antidepressants in elderly patients for non-depressive indications (including off-label) such as anxiety disorders, insomnia, pain, panic disorders (with or without agoraphobia), fibromyalgia, obsessive-compulsive disorder, social phobia, nicotine dependence, attention deficit hyperactivity disorder, posttraumatic stress disorder, digestive system disorders, urinary system disorders, bulimia nervosa, … This workshop will give an overview of the literature and will include clinical advise based on current guidelines.

### W033

### Important pharmacokinetic and pharmacodynamic considerations for dose changes of antipsychotics and antidepressants

M. Stuhec

Pharmacokinetic and pharmacodynamic considerations of antipsychotic and antidepressant treatment is one of the key aspects of medication dosing. These medications are very different in terms of pharmacokinetic and pharmacodynamic profiles that can affect dosing and therefore this knowledge is necessary to manage these problems in real clinical practice. In addition, many trials and meta-analyses including older adults have been conducted without these very important considerations; however patients with these problems are the most frequent in real clinical practice and are frequently treated with multiple medications (polypharmacy) and have high comorbidities and drug-drug interactions. Most problems could be avoided by adopting appropriate treatment strategies (e.g. tools) including pharmacokinetic tools, collaborative care, and different medication lists.
In this workshop we will present firstly a pharmacokinetic and a pharmacodynamic overview of medication dosing and possible tools to reduce problems in these areas. The main focus of this talk will be on treatment hints which could be used in real clinical practice to reduce these problems and their clinical consequences in older adults.

## Responding to violence against women as clinicians: where do legal aspects fit in?

### W040

### Violence against women in Russia and preventive measures: looking back, moving forward

N. Semenova

In February 2017, Russia decriminalized domestic violence in cases where it does not cause "substantial bodily harm" and does not happen more than once a year. The independent media outlets have published a joint study on violence against women in Russia. Nearly four in five women convicted of murder in Russia have survived domestic violence. According to Russian police statistics, 15.381 women suffered domestic violence in the first nine months of 2019. This paper explores the common concerns expressed by experts, gives an overview of some cases (Khachaturian sisters' case – three teenage sisters stabbed and battered their father to death, the girls' father abused them physically and psychologically for years; the case of Margarita Gracheva – the 27-year-old mother of two boys, the husband chopped off her hands; and Ms. Yeshchenko case – the lover and former student of a historian who murdering her and dismembering her body) and provides strategies for prevention. This paper uses these endeavors as a case study with which to explore the perceived responsibilities of psychiatrists, to comment on and respond to the broader social and political context. This paper is a multi-stakeholder exploration of the position of my own with regard to the way preventive issues were being handled within the context of changing practices, policy, legislation and attitudes. The lessons learned to date the tricks and the pitfalls will be discussed.

## The schizophrenia label: patient, family and professional perspectives

### W049

### The name "schizophrenia": initiatives and proposals from the national psychiatric associations

S. Vahip

Renaming schizophrenia which started from Japan has received heterogeneous reactions from all over the world. Some countries, associations and experts consider it as an opportunity to fight against stigma while others do not share this perspective. Reactions, initiatives and proposals collected from EPA (European Psychiatric Association) member National Psychiatric Associations (NPAs) will be summarized to facilitate the discussion. A survey, consisting of structured and semi-structured questions was sent to 44 EPA member NPAs from 40 countries. Main purpose of the survey was to search and collect the NPAs' opinions on several perspectives including any previous or planned action for this issue, possible prediction on whether renaming could be effective on stigmatization in their countries and cultures, and any proposals as possible new names for schizophrenia. Also, NPAs had been requested to provide evaluations of their Psychosis or Schizophrenia Section members, or other experts from their countries via the same instrument. The collected data will be presented in this workshop to facilitate the discussion and to elaborate Europe-wide or country specific positions and needs on the issue.

### W050

### Changing the name of schizophrenia: international challenges and future perspectives

W. Gaebel

Many patients, clinicians, and researchers consider the label schizophrenia to be stigmatizing. Thus, over the last years, a name change has increasingly been discussed to relieve patients from the negative beliefs, prejudice, and discrimination associated with the label schizophrenia (Gaebel & Kerst, 2019). In different world regions, stakeholders have developed alternative labels for schizophrenia. For example, some Asian countries have implemented alternative names in their respective healthcare systems. Also in Europe, different alternative labels have been proposed (e.g., salience dysregulation syndrome), but none have, thus far, been systematically implemented in mental healthcare. In sum, to date, there is no commonly agreed upon alternative label or conceptualization to replace schizophrenia internationally. Meeting relevant criteria for a new name, finding agreement among all involved stakeholders and replacing the established term is a complex process. This effort would be worthwhile if it contributes to a reduction of stigmatization and an improvement in mental healthcare for patients with schizophrenia. Thus far, however, there is only limited empirical evidence that a name change alone will achieve the desired effects. Hence, it may be premature to abandon the concept of schizophrenia, which has proven its reliability, clinical utility, and validity over the last century. In this lecture, the challenges, potential risks, and potential advantages of developing an internationally consistent alternative label for schizophrenia will be discussed. Gaebel, W., & Kerst, A. (2019). The debate about renaming schizophrenia: a new name would not resolve the stigma. Epidemiology and Psychiatric Sciences, 28(3), 258-261.

## An exciting time for research for early career psychiatrists: methods and perspectives

### W058

### Worldwide network web-based transcultural research

V. Pereira-Sanchez[1,2]

Current early-career psychiatrists (ECPs) were grown and trained in the internet era, and web-based tools have expanded their international research possibilities. As real-time communication across the world has been enabled, ECPs can start and expand multicentric research studies with transcultural and multidisciplinary perspectives of many colleagues from developed and developing countries. This research could be much better in sample sizes and impact than small studies in single locations; furthermore, many ECPs may find it easier to join and lead web-based research teams that in their local institutions. However, these large and heterogeneous teams face unique challenges when aiming at coordinating effectively their efforts and translating them into high-impact scientific publications. This talk will discuss these opportunities and challenges, with a practical viewpoint, offering examples of accessible internet-based tools and successful and ongoing research studies. The speaker has participated in and leaded studies of this such and aims to enhance a discussion among listeners on how to further promote and improve this kind of research.

**Conflicts of Interest:** No

### W060

### When one study is not enough: systematic reviews?

O. Kilic

A systematic review is a research article that identifies a specific review question, identifies all relevant studies, appraises their quality, and summarizes the results of all high-quality research evidence using a scientific methodology. High-quality research includes those studies with an explicit and rigorous design and those that answer a given research question. Understanding of systematic reviews and implementing them in practice is becoming essential for all healthcare professionals. However, before using the results, it is crucial to appraise a systematic review accurately by asking a series of questions to evaluate whether the review conducted all the steps correctly with minimal bias. Several steps, when conducting a systematic review, will be covered in this presentation. 1. Asking an answerable and focused review question 2. Writing the plan and background to the review 3. Specifying objectives and inclusion and exclusion criteria 4. Conducting a comprehensive and systematic literature search 5. Working with primary papers: selecting, appraising and extracting data 6. Synthesizing, summarizing and presenting findings 7. Writing up discussion and completion review 8. Sharing, disseminating, and using systematic reviews to inform and improve practice.

### W062

### The exposome research paradigm in psychiatry

S. Guloksuz

Several environmental exposures have been associated with mental disorders: childhood adversities, stressful life events, drug and alcohol use, obstetric and pregnancy complications, urban environment, and ethnic minority status. Research has thus far investigated the association between a single psychiatric outcome and a single environmental exposure, such as childhood adversities and psychosis.

However, exposures come in dense networks of closely related and interactive elements. The exposome, the totality of the environmental exposures (nongenetic component) from the prenatal period onwards, consists of dynamic, interactive, and intertwined categories that include the internal (e.g., gut microflora), the specific external (e.g., drug use), and the general external (e.g., urban environment). The exposome approach offers a holistic understanding of environment, similar to the genome concept, and provides a theoretical framework to investigate the role of the poly-gene and poly-environment in mental health. Guided by the exposome paradigm, we showed how predictive modeling approaches could be applied to construct an exposome score (ES) for schizophrenia, which is a single metric of aggregated environmental load, similar to polygenic risk score. In a series of research, we further demonstrated the utility of the ES for schizophrenia for estimating environmental load, testing gene-environment interactions, and stratification in independent datasets. Galvanized by the availability of growing amounts of multimodal, multilevel, multidimensional data, along with the accessible advanced statistical learning methods to handle high-dimensional data, exposome research is anticipated to the catch up with the pace of progress in the psychiatric genomics.

### How we can achieve. the impossible in psychiatry: personal stories, perspectives and lessons for the future

#### W067

#### Reflections on the role of women in psychiatry: a personal journey

D. Wasserman

The ability to determine the direction of one's work, is one of the important goals in my working life. To achieve it, a mentorship and role models of female teachers and scientists in all stages of my education alongside with many other male role models were important for my personal and career development. Also, to learn how very negative life events and key obstacles can be overcome when being aware of the long-time perspective and the context one lives in.

**Conflicts of Interest:** No
**Keywords:** Role models

### What I wish I would have learned earlier about the management of borderline personality disorder

#### W076

#### What I explain to my patients about BPD diagnosis

L. De Picker

**Introduction:** Borderline personality disorder (BPD) is very common, with an estimated community prevalence of 1-3% and up to 25% in psychiatric inpatients. Most early career psychiatrists indicate they feel ill-equipped to deal with this important patient group. BPD patients may present with specific needs and disruptive symptoms which can be challenging for unexperienced professionals, while psychiatric training often does not offer the necessary knowledge and skills.
**Objective:** Scientific evidence points out that for most BPD patients a pragmatic 'good enough' psychiatric management will suffice (Choi-Kain and Gunderson 2019) - even if clinicians did not receive specialized psychotherapeutic training. One of the key interventions in the management of BPD patients is adequate diagnostic disclosure.
**Results:** BPD patients are often withheld diagnostic information or diagnosed instead with depression or anxiety disorders which are often comorbid but rarely the primary reason for hospital admission. Consequently, the solution is inappropriately framed as external (for instance medication) rather than internal. Diagnostic disclosure also offers an excellent opportunity for psychoeducation: a review of diagnostic criteria should always be accompanied by a narrative explanation using a validating model. Psychoeducation should emphasize three key points: (1) that trait-factor emotional vulnerability or interpersonal hypersensitivity is heritable; (2) that most BPD patients remit and (3) that BPD is a treatable condition.
**Conclusion:** BPD is a treatable condition and good psychiatric management for this disorder is sufficient for most patients. Adequate diagnostic disclosure and psychoeducation is a key intervention which improves patients' hope, trust and compliance with treatment.

#### W077

#### Help! is there a psychotherapist in the room?

L. Egervári

**Introduction:** Borderline personality disorder (BPD) is very common, with an estimated prevalence of 1-3% in the community, rising to 25% in psychiatric inpatients. Yet most early career psychiatrists indicate they feel ill-equipped to deal with this important patient group. BPD patients may present with specific needs and disruptive symptoms which can be challenging for unexperienced professionals, while psychiatric training often does not offer the necessary knowledge and skills.
**Objective:** Scientific evidence points out that for most BPD patients, a pragmatic 'good enough' psychiatric management will suffice – implying clinicians need not refrain from treating these patients even if they did not receive specialized psychotherapeutic training. We will discuss the most important principles which can guide clinicians in their approach of BPD patients, with an emphasis on psychotherapeutic approaches in this part of the workshop.
**Results:** There are psychotherapeutic methods designed specifically to help BPD patients, such as dialectical behavior therapy, schema therapy, mentalization based therapy and transference-focused psychotherapy. In the workshop we are going to discuss the theoretical and practical aspects of these treatments. Most importantly though, psychotherapy offers insights about how to address patients with BPD regardless of treatment setting. In the workshop we are going to demonstrate and practice basic techniques which can be useful when treating BPD patients.

**Conclusion:** One does not need to be a fully trained specialized psychotherapist in order to work with BPD patients in a meaningful way, good psychiatric management for this disorder is sufficient for most patients.

### W078

### When and why you should (not) use drugs in the treatment of BPD

T. Gondek

**Introduction:** Borderline personality disorder (BPD) is very common, with an estimated prevalence of 1-3% in the community, rising to 25% in psychiatric inpatients. Yet most early career psychiatrists indicate they feel ill-equipped to deal with this important patient group. BPD patients may present with specific needs and disruptive symptoms which can be challenging for unexperienced professionals, while psychiatric training often does not offer the necessary knowledge and skills.

**Objective:** With limited access to practical psychotherapeutic training during the residency in psychiatry in many European countries, early career psychiatrists might routinely resort to prescribing medications to these patients, particularly in inpatient setting, despite scarce evidence for such interventions. The aim of the presentation is to discuss the existing guidelines and current body of knowledge about pharmacological management of patients with BPD.

**Results:** Psychotherapy, such as dialectical behavior therapy, schema therapy, mentalization based therapy and transference-focused psychotherapy, remain the treatment of choice for BPD patients. There is no evidence pharmacotherapy alone may be effective, however a combination of both kinds of treatments may be considered in the management of specific symptoms of this personality disorder, while it can also be useful in psychiatric comorbidities. Antidepressants, mood stabilizers and antipsychotics are among the most common prescribed medications for BPD patients

**Conclusion:** Pharmacological interventions are not approved in the treatment of BPD and will not be sufficient in the management of BPD, yet are frequently used to manage also some of its core symptoms. Polypharmacy in these patients is discouraged.

**Disclosure:** In the past 12 months I received honoraria from Lundbeck Poland and (indirectly) Aurovitas Pharma Polska (Apotex Poland).

## Aggressiveness and violent behaviours in psychiatric settings

### W082

### Breaking the law: correlates of aggressiveness and violent crimes in bipolar disorders

A. Murru

The relationship between aggressiveness and violence (A/V) in psychiatric disorders is an enduring topic where strong opinions tend to stem far beyond scientific evidence. Unluckily, mass media often tend to emphasize mental illness as the leading cause of violence in mass-shootings or terrorist attacks, and cinematographic depictions of the mentally ill are often unrealistically sensationalized, contributing to stigma concerning psychiatric conditions. Bipolar disorder (BD) is is a chronic recurring condition characterized, among other aspects, by wide variations in mood and energy levels. The clinically frequent co-occurrence of increased impulsivity with mood and energy activation leads to an unmediated, automated cliché of increased rates of aggressiveness or violence (A-V). This presentation will focus on the clinical correlates of A/V in BD as a paradigm and pitfall of current research concerning A/V in psychiatric disorders. Aspects discussed will outline the possible factors, or lack of, predisposing to A/V in clinical settings. Implications of the possible presence of A/V on patients' course of illness, functioning and quality of life will be discussed, as well as possible multidisciplinary approaches that may alleviate the impact of A/V on both patients and caregivers

## Growing old: strategies for an appropriate clinical management of mental health issues of persons with intellectual disabilities

### W086

### A new challenge? the mental health of elderly people with intellectual disabilities

M.-M. Theil

**Introduction:** Intellectual disability (ID) is a neurodevelopmental disorder of etiologically diverse conditions starting in childhood characterised by impairments in intellectual and adaptive functioning. Although life expectancy is still lower compared with the general population, the number of people with ID growing old is increasing. Consequently, age-related mental health (MH) disorders, such as dementia, are becoming a common comorbidity of ID.
**Objectives:** Description of the challenges related to the provision of appropriate healthcare for elderly persons with ID and MH disorders.
**Method:** Review of the recent literature based upon a comprehensive search of medical databases using the keywords: intellectual disability, mental health, dementia, Alzheimer's disease, Down's syndrome and age.
**Results:** ID is associated with a higher prevalence of physical and MH disorders across all ages, while clinical diagnostics and management of dementia are made complicated by the comorbidity rate. Elderly people with ID, especially those with Down's syndrome (DS), more commonly develop dementia than their age-matched counterparts without ID. For people with DS and Alzheimer's disease (AD) the survival time was related to the age at diagnosis of AD, severity of ID, anti-dementia medication, history of epilepsy and living status.
**Conclusion:** Elderly people with ID have greater early-onset multimorbidity and a prevalence of dementia which is up to five times higher compared to the elderly non-ID population. Their care is more challenging, and their diagnostics requires a comprehensive approach, including ID-appropriate neuropsychological evaluation. Specialist services for elderly persons with ID and MH disorders are, however, sparsely available and should be established.

### W088

### Getting older with intellectual disabilities: clinical perspective on mental health and cognitive dysfunction progression

T. Sobow

Elderly people with intellectual disabilities represent one of the fastest growing groups of the special needs populations. This is mainly the consequence of improved and adaptive care standards as well as better general medical treatments. Living longer also carries more mental health problems, including those related to cognitive dysfunction and dementia. Such patients are often treated with antipsychotics and mood stabilizers, with main reason to use of these drugs being „sedation". At least some of them may benefit form cholinesterase inhibitors and / or memantine, although evidence form clinical trials is scarce. Also antidepressants, particularly those blocking serotonin and / or norepinephrine transporters are probably underused. Importantly, understanding behavioral and psychological problem of these patients within the frame of the biopsychosocial paradigm might be useful, leading to potentially effective and harmless non-pharmacological interventions. Particularly, the concepts of attention to frustrated needs and reactions to environmental change may prove to be fruitful.

### W090

### Diagnostics of dementia in individuals with intellectual disabilities using neuropsychological methods

C. Krzoska

**Introduction:** Individuals with intellectual disabilities (ID) are increasingly living to old age, and are thus affected by age-related diseases, such as dementia. The diagnosis of dementia in individuals with ID requires suitable, population-specific assessment tools, as standard neuropsychological assessment instruments are often not appropriate for individuals with ID.
**Objectives:** Analysis of specific neuropsychological assessment instruments for the diagnosis of dementia in individuals with ID.
**Method:** Comparison of the common neuropsychological assessment instruments for individuals with ID (DMR, DSQIID, DSDS and DTIM) based upon a comprehensive review of the literature.
**Results:** All instruments can detect dementia-specific symptoms. The majority of neuropsychological assessment instruments are designed as informant interviews. These have inherent methodological problems concerning inter-rater and test-retest reliability, which must be taken into account when interpreting the results. The DTIM addresses this problem by combining an informant interview with a neuropsychological assessment.
**Conclusion:** Diagnosing dementia in individuals with ID is especially difficult and complex, as functional capacities commonly impaired by dementia are likely to be already compromised by ID. A further issue is patient cooperation during testing. Neuropsychological assessments have a range of advantages in the diagnostic process. They are non-invasive and offer valuable information even when there is only early suspicion of dementia. Furthermore, neuropsychological assessments are capable of determining individual functional capacities, which is essential for the development and provision of patient-specific dementia care plans. Besides other common neuropsychological assessment instruments, individuals with ID benefit particularly from the DTIM due to its special design.

### W091

### Support for caregivers of adults with intellectual disabilities suffering from early-onset dementia

K. Krysta

The care for persons with intellectual disability is usually an effort taken by their parents or other members of the family. The amount of time and devotion to this task depends highly on the level of the disability. At different phases of life of the person with intellectual disability the care is shared with different institutions like rehabilitation centers, special schools, vocational workshops, occupational activity centres etc. However, at the older age the health problems may increase, including the development of early dementia, which makes the participation in the above programs no longer possible. This causes that the responsibility of the caregivers increases, which requires still more efforts from them. This situation is complicated with the fact that the caregivers themselves grow older and they often need a support for themselves. Described here is a case of a female patient suffering from Down syndrome, who used to participate in a program of a vocational training workshop for many years, which allowed her for social integration and acquiring news skills. However, as she grew older, the signs of early dementia appeared, which were later accompanied with neurological symptoms, which made walking no longer possible for her. As she became unable to participate in the accessible rehabilitation programs anymore, she had to stay at home with her mother. The possible ways to support and assist her mother as a caregiver in this situation are discussed.

### The value of treatment for eating disorders: a joint EPA & EBC project

#### W092

#### Care pathways overview. Improving transitions from inpatient care: collaborative digital solutions

J. Treasure

Background. Inpatient care for anorexia nervosa is used for patients at high medical and psychiatric risk and who cannot be safely managed as outpatients. Approximately a third of patients relapse within the first 6 months. The aim of this programme of work was to examine whether psychoeducational interventions preparing for the transition back to the community codesigned by, and for, carers and patients to carers are of benefit. Methods Study 1 (CASIS). Patients (n=178) admitted to inpatient units across the UK entered a randomised trial in which carers received treatment as usual, with, or without augmentation with carers self-management materials. Outcomes were followed over two years (Hibbs et al 2016, Magill et al 2017). Study 2 (TRIANGLE pilot) Patient (n=31) and carers (n=21) admitted to inpatient care were given the ECHOMANTRA intervention (with separate subsections for each) and were followed for 3 months after discharge (Adamson et al 2019). Results In CASIS, carer burden was reduced (Effect size 0.5) as was carer emotional behaviour (Effect size 0.5). The length of admission was shortened (148 vs 168 days) and re-admission rates were reduced (27% vs 32%; p=0.04). In the TRIANGLE pilot there were positive comments about the collaborative work from both parties. The length of admission was reduced by 4.5 weeks and weight gain was increased by 0.11 kg/week in the group given the ECHOMANTRA intervention in comparison to outcomes from audit data. Conclusion Augmenting treatment to help patients and carers to prepare for discharge can consolidate changes from inpatient care.


Article ID: W004
Subjects: Abstract; Acute Short-Lived Psychotic Disorders: Clinical, Nosological and Socio-Cultural Aspects

Clinical, demographic characteristics and outcome of acute and transient psychotic disorder in Ibadan, Nigeria

Esan O.
University of Ibadan , Nigeria, Department of Psychiatry, Ibadan , Nigeria
Keywords: outcome, Ibadan, Nigeria, Clinical and Demographic characteristics, Acute and Transient Psychotic Disorder

Conflicts of Interest:

No

Article ID: W009
Subjects: Abstract, Early intervention/early detection in psychosis in real-life practice: different models across Europe

Implementing early detection of psychosis in France: the potential of adolescents’ houses

Laprevote V. 1 *
Mignot T. 2
Bernardin F. 1
Schwan R. 3
1 Centre Psychothérapique de Nancy, Clip, Pôle Hospitalo-universitaire De Psychiatrie D’adultes Du Grand Nancy , Nancy , France
2 Centre Psychothérapique de Nancy, Clip (centre De Liaison Et D'intervention Précoce), Nancy , France
3 Centre Psychothérapique de Nancy, Pôle Hospitalo-universitaire De Psychiatrie D’adultes Du Grand Nancy, nancy , France
* Corresponding author.
Keywords: early detection, psychosis, Early intervention

Conflicts of Interest:

No

Article ID: W012

First episode psychosis integrative treatment: a 20-year experience in Tallinn, Estonia

Peebo K.
The North Estonia Medical Centre, First-episode Psychosis Integrative Treatment Department , Tallinn , Estonia
Keywords: First Episode Psychosis, estonia, FEP treatment

Conflicts of Interest:

No

Article ID: W023
Subjects: Abstract, Assisted Suicide in the Elderly

Media and suicidal behaviour

Hegerl U. 1 2
1 Goethe University Frankfurt , Department of Psychiatry, Psychosomatics, And Psychotherapy, Frankfurt a.M. , Germany
2 Goethe University , Deutsche Depressionshilfe, Leipzig , Germany
Keywords: Social Media, werther effect, Suicide prevention

Conflicts of Interest:

No

Article ID: W024
Subjects: Abstract, Psychotherapy training in Europe

Trainees are motivated but have difficulty to find resources

Gargot T.
Pitie Salpetriere Hospital, Child and Adolescent Psychiatry, Charenton le Pont , France
Keywords: psychotherapy training, survey, European Federation of Psychiatric Trainees

Conflicts of Interest:

No

Article ID: W026

EFPT psychotherapy guidebook: the story

Koutsomitros T.
Aristotle University of Thessaloniki , 2nd Department of Psychiatry, Thessaloniki , Greece
Keywords: EFPT: European Federation of Psychiatric Trainees, Psychotherapy training in Europe, psychotherapy, training in psychotherapy

Conflicts of Interest:

No

Article ID: W028

EPA online course about motivational interviewing: present and future perspective

Browne P.
Gloucestershire Health and Care NHS Foundation Trust, Wotton Lawn Hospital , Gloucester , United Kingdom
Keywords: motivational interviewing, online course

Conflicts of Interest:

No

Article ID: W029
Subjects: Abstract, Antipsychotics and antidepressants dosing. Focus on elderly patients

Recommendations for dosing antipsychotics in schizophrenia

Bitter I.
Semmelweis University , Department of Psychiatry and Psychotherapy, Budapest , Hungary
Keywords: antipsychotics, schizophrenia, treatment, dose

Article ID: W032

The use of antidepressants in elderly patients with various psychiatric disorders

Bouckaert F.
University Psychiatric Center KU Leuven & Laboratory for Translational Neuropsychiatry , Department for Psychiatry, Leuven , Belgium
Keywords: Dépression, off label, Antidepressants, Elderly

Conflicts of Interest:

No

Article ID: W033

Important pharmacokinetic and pharmacodynamic considerations for dose changes of antipsychotics and antidepressants

Stuhec M.
University of Ljubljana , Faculty of Pharmacy & Ormoz Psychiatric Hospital, Clinical Pharmacy, Ormoz, Slovenia
Keywords: pharmacokinetics, Pharmacodynamics, Dosing, psychopharmacology

Conflicts of Interest:

No

Article ID: W040
Subjects: Abstract, Responding to Violence against Women as Clinicians: Where Do Legal Aspects Fit In?

Violence against women in Russia and preventive measures: looking back, moving forward

Semenova N.
Moscow Research Institute of Psychiatry – a branch of V. Serbsky National Medical Research Centre for Psychiatry and Narcology , Moscow, Russia, Psychiatry, Moscow , Russian Federation
Keywords: violence against women, Russia, preventive measures, decriminalization

Conflicts of Interest:

No

Article ID: W049
Subjects: Abstract; The Schizophrenia Label: Patient, Family and Professional Perspectives

The name “schizophrenia”: initiatives and proposals from the national psychiatric associations

Vahip S.
Ege University Medicine Faculty , Department of Psychiatry, Izmir , Turkey
Keywords: renaming, schizophrenia, National Psychiatric Associations, stigmatization

Conflicts of Interest:

No

Article ID: W050

Changing the name of schizophrenia: international challenges and future perspectives

Gaebel W.
Heinrich-Heine-Universität Düsseldorf, Department of Psychiatry and Psychotherapy, Lvr Klinikum , Düsseldorf , Germany
Keywords: schizophrenia, renaming, Stigma

Conflicts of Interest:

No

Article ID: W058
Subjects: Abstract, An Exciting Time for Research for Early Career Psychiatrists: Methods and Perspectives

Worldwide network web-based transcultural research

Pereira-Sanchez V. 1 2
1 Clinica Universidad de Navarra, Psychiatry and Medical Psychology , Pamplona , Spain
2 Hassenfeld Children’s Hospital at NYU Langone, Department of Child And Adolescent Psychiatry , New York , United States of America

Conflicts of Interest:

No

Article ID: W060

When one study is not enough: systematic reviews?

Kilic O.
Koç University Hospital , Department of Psychiatry, Istanbul , Turkey
Keywords: Systematic Review

Conflicts of Interest:

No

Article ID: W062

The exposome research paradigm in psychiatry

Guloksuz S.
Maastricht University Medical Center , Department of Psychiatry and Neuropsychopharmacology, Maastricht , Netherlands
Keywords: Risk score, Environment, Predictive modeling, psychosis

Conflicts of Interest:

No

Article ID: W067
Subjects: Abstract; How We Can Achieve. the Impossible in Psychiatry: Personal Stories, Perspectives and Lessons for the Future

Reflections on the role of women in psychiatry: a personal journey

Wasserman D.
Karolinska Institute , National Centre For Suicide Research and Prevention of Mental Lll-health, Stockholm , Sweden
Keywords: Role models

Conflicts of Interest:

No

Article ID: W076
Subjects: Abstract, What I wish I would have learned earlier about the management of borderline personality disorder

What I explain to my patients about BPD diagnosis

De Picker L.
University Psychiatric Hospital Campus Duffel , Sinaps, Duffel , Belgium
Keywords: psychoeducation, good psychiatric management, Borderline Personality Disorder

Conflicts of Interest:

No

Article ID: W077

Help! is there a psychotherapist in the room?

Egervári L.
Semmelweis University , Department of Psychiatry and Psychotherapy, Budapest , Hungary
Keywords: early career, Borderline Personality Disorder, psychotherapy

Conflicts of Interest:

No

Article ID: W078

When and why you should (not) use drugs in the treatment of BPD

Gondek T.
European Psychiatric Association, Early Career Psychiatrists Committee, Wroclaw , Poland
Keywords: Borderline Personality Disorder, treatment, pharmacotherapy, management

Article ID: W082
Subjects: Abstract, Aggressiveness and Violent Behaviours in Psychiatric Settings

Breaking the law: correlates of aggressiveness and violent crimes in bipolar disorders

Murru A.
Bipolar and Depressive Disorders Unit, Hospital Clínic de Barcelona, Psychiatry , Barcelona , Spain
Keywords: aggressiveness, Bipolar disorder, crime

Conflicts of Interest:

No

Article ID: W086
Subjects: Abstract, Growing Old: Strategies for an Appropriate Clinical Management of Mental Health Issues of Persons with Intellectual Disabilities

A new challenge? the mental health of elderly people with intellectual disabilities

Theil M.-M.
Evangelische Stiftung Neuerkerode, Lukas-Werk Gesundheitsdienste GmbH, Integrierter Gesundheitsdienst Neuerkerode And Mzeb Braunschweig, Sickte-Neuerkerode , Germany
Keywords: mental health, dementia, age, Intellectual disability

Conflicts of Interest:

No

Article ID: W088

Getting older with intellectual disabilities: clinical perspective on mental health and cognitive dysfunction progression

Sobow T.
Dialog Therapy Centre, Psychiatry , Warsaw , Poland
Keywords: intellectual disability, elderly, mental health, cognitive dysfunction

Conflicts of Interest:

No

Article ID: W090

Diagnostics of dementia in individuals with intellectual disabilities using neuropsychological methods

Krzoska C.
Evangelische Stiftung Neuerkerode, Lukas-Werk Gesundheitsdienste GmbH, Integrierter Gesundheitsdienst Neuerkerode And Mzeb Braunschweig, Sickte-Neuerkerode , Germany
Keywords: Intellectual disability, dementia, neuropsychological assessment, mental health

Conflicts of Interest:

No

Article ID: W091

Support for caregivers of adults with intellectual disabilities suffering from early-onset dementia

Krysta K.
Medical University of Silesia in Katowice , Department of Rehabilitation Psychiatry, Katowice , Poland
Keywords: Caregivers, Down Syndrome, Intellectual disability, dementia

Conflicts of Interest:

No

Article ID: W092
Subjects: Abstract, The value of treatment for eating disorders: a joint EPA & EBC project

Care pathways overview. Improving transitions from inpatient care: collaborative digital solutions

Treasure J.
King’s College, Institute of Psychiatry , London , United Kingdom
Keywords: Anorexia nervosa, carer, inpatient

Conflicts of Interest:

No

